# Cervical Tuberculous Lymphadenitis Associated With New-Onset Acanthosis Nigricans Following Antitubercular Therapy: A Case Report

**DOI:** 10.7759/cureus.65012

**Published:** 2024-07-20

**Authors:** Jimmy Meade, Warren Back, Salman Arif, Claudiu Georgescu, Jianlin Tang, Michael Ellis

**Affiliations:** 1 Division of Infectious Diseases, The University of Toledo College of Medicine and Life Sciences, Toledo, USA; 2 Department of Surgery, The University of Toledo College of Medicine and Life Sciences, Toledo, USA

**Keywords:** side effects of medical treatment, tuberculosis therapy, scrofula, acanthosis nigricans, ripe, cervical lymphadenitis, tb – tuberculosis

## Abstract

Cervical tuberculous lymphadenitis (CTL), also known as scrofula, is an extrapulmonary manifestation of tuberculosis, a disease that is endemic to many developing countries, particularly Sub-Saharan Africa and Asia, but may also be found worldwide in developed countries like the United States. CTL can be difficult to detect and may mimic other similar-appearing conditions, so a high index of suspicion is required to accurately diagnose the condition when a patient presents with one or more neck masses. Incision and drainage and excisional surgery are aggressive options available to treat CTL but are not preferred due to a high risk of serious adverse events like fistulization and hematological dissemination. Clinicians typically opt for traditional tubercular RIPE (rifampin, isoniazid, pyrazinamide, and ethambutol) therapy for its high efficacy in treating extrapulmonary tuberculosis. Despite this preference, RIPE therapy has been known to elicit a myriad of side effects that demand close monitoring by clinicians. One side effect of the RIPE regimen that has yet to be reported is acanthosis nigricans (AN), a dermatological sign that presents as thickening and darkening of the skin, often in intertriginous areas. AN frequently occurs in conjunction with insulin resistance, and interestingly, the RIPE drug isoniazid has been implicated in insulin derangements in patients, most notably diabetics. However, the incidence of AN secondary to isoniazid use has not been explicitly recorded in the literature to date. Herein we present a novel case of a young man from Nepal with CTL treated via RIPE therapy who developed AN likely secondary to isoniazid use.

## Introduction

Tuberculosis (TB) is a disease that still has significant morbidity and mortality worldwide, with 10 million people suffering from TB-related illnesses yearly and 1.3 million deaths occurring in 2022 [1]. TB remains a persistent global health threat due to diverse clinical presentations, diagnostic intricacies, lack of access to resources, and increased prevalence of drug-resistant strains of TB [1,2]. Cervical tuberculous lymphadenitis (CTL), also known as scrofula, is a manifestation of extrapulmonary TB and poses a considerable health challenge, particularly in regions with a high burden of TB [3]. Tuberculous lymphadenitis, characterized by lymph node involvement, often mimics other granulomatous or neoplastic conditions [4]. Late diagnosis may lead to multiple complications [3]. Once appropriately identified, the condition can be easily treated; however, multiple side effects may result from this treatment.

Acanthosis nigricans (AN) is a cutaneous disorder characterized by dark, velvety plaques most commonly occurring around skin folds in areas such as the axilla, neck, breast, and groin [5]. AN often occurs with coexisting obesity and diabetes and therefore may be indicative of underlying metabolic disruption [5]. It is treatable with conservative measures that address the underlying cause such as weight loss, stopping an offending agent, and using systemic or topical treatments [5].

Through this case report, we highlight not only the diagnostic journey of CTL but also the multifaceted approach required for successful therapeutic outcomes. We provide a comprehensive overview of a clinical case, focusing on the presentation of CTL, diagnosis, management, treatment side effects, and patient prognosis. A thorough understanding of this potential presentation is crucial for clinicians to recognize and manage extrapulmonary TB with atypical responses to standard treatment.

## Case presentation

**Figure 1:**
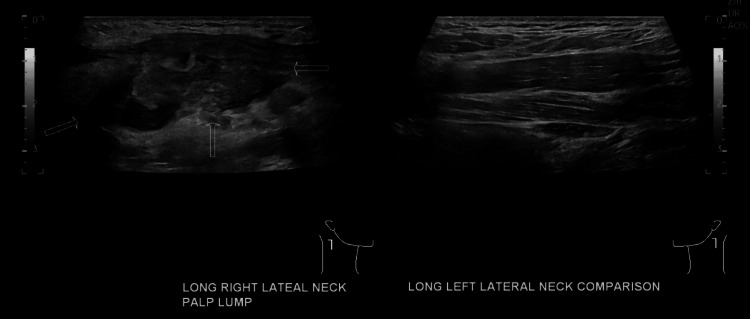
Ultrasound comparing both sides of the neck. Arrows indicate lesion on the right neck.

A 27-year-old male with no significant past medical or surgical history, originally from Nepal, presented with a chief complaint of a progressively enlarging neck lump accompanied by fatigue and a low-grade fever. He had recently returned from Nepal three weeks prior, where he underwent dental procedures. Shortly after his return, he noticed the lesion, prompting his initial visit to primary care. Upon examination, the patient was found to have a 3 cm by 4 cm slightly erythematous, tender mass on the right neck and a temperature of 100.6°F. A course of Augmentin was prescribed for a suspected infectious etiology. Other than slightly elevated liver enzymes (aspartate transaminase [AST] 44, alanine transaminase [ALT] 83), complete blood count (CBC) and comprehensive metabolic panel (CMP) were unremarkable and Epstein-Barr virus screening serology was negative. Ultrasound demonstrated a hypoechoic heterogeneous mass suggestive of pathologic adenopathy versus primary tumor and prominent lymph nodes (Figure 1).

**Figure 2:**
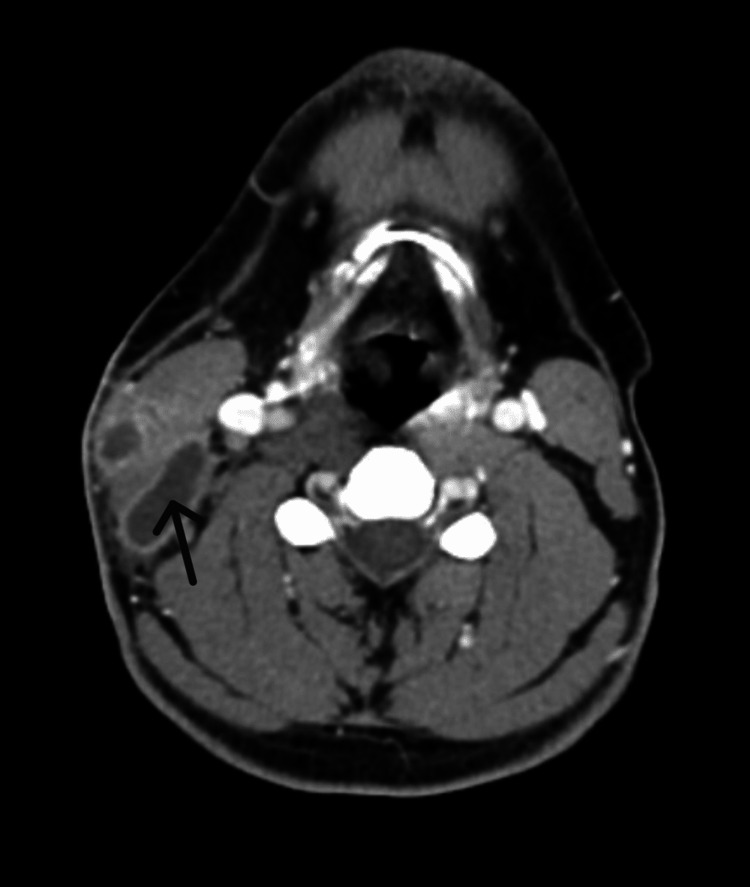
CT soft tissue neck with IV contrast

At follow-up two weeks later, the lump persisted in size and became more painful with no resolution of fatigue. Additional investigations, including a thyroid panel, HbA1c, and infectious screenings (HIV, rapid plasma reagin [RPR], chlamydia/gonorrhea, hepatitis B) were within normal limits. The lipid panel demonstrated mild elevation of triglycerides, cholesterol, and very low-density lipoprotein. The patient was referred to general surgery where 1 mL of yellow pus was drained and sent for acid-fast bacilli (AFB) culture and sensitivity. He was then referred to the Infectious Diseases clinic, where the mass was noted by the patient to be slightly smaller but remained tender. A computerized tomography (CT) scan of the neck with IV contrast showed a complex mass/fluid collection in the right neck (Figure 2). CT scans of the chest, abdomen, and pelvis with IV contrast were all negative for additional masses or lymphadenopathy.

**Figure 3:**
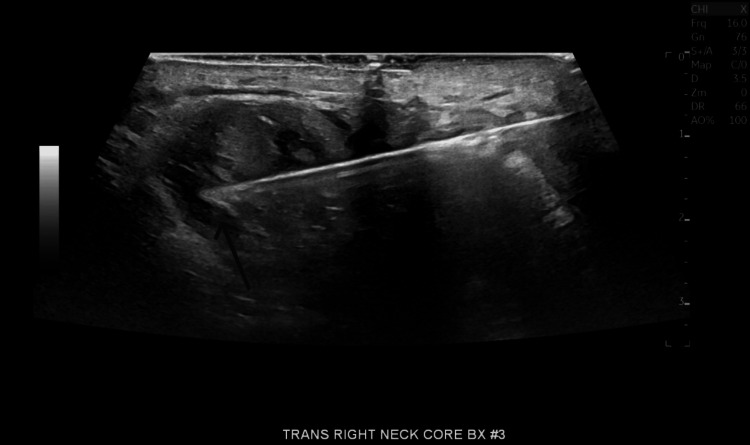
Ultrasound-guided biopsy of neck lump

**Figure 4:**
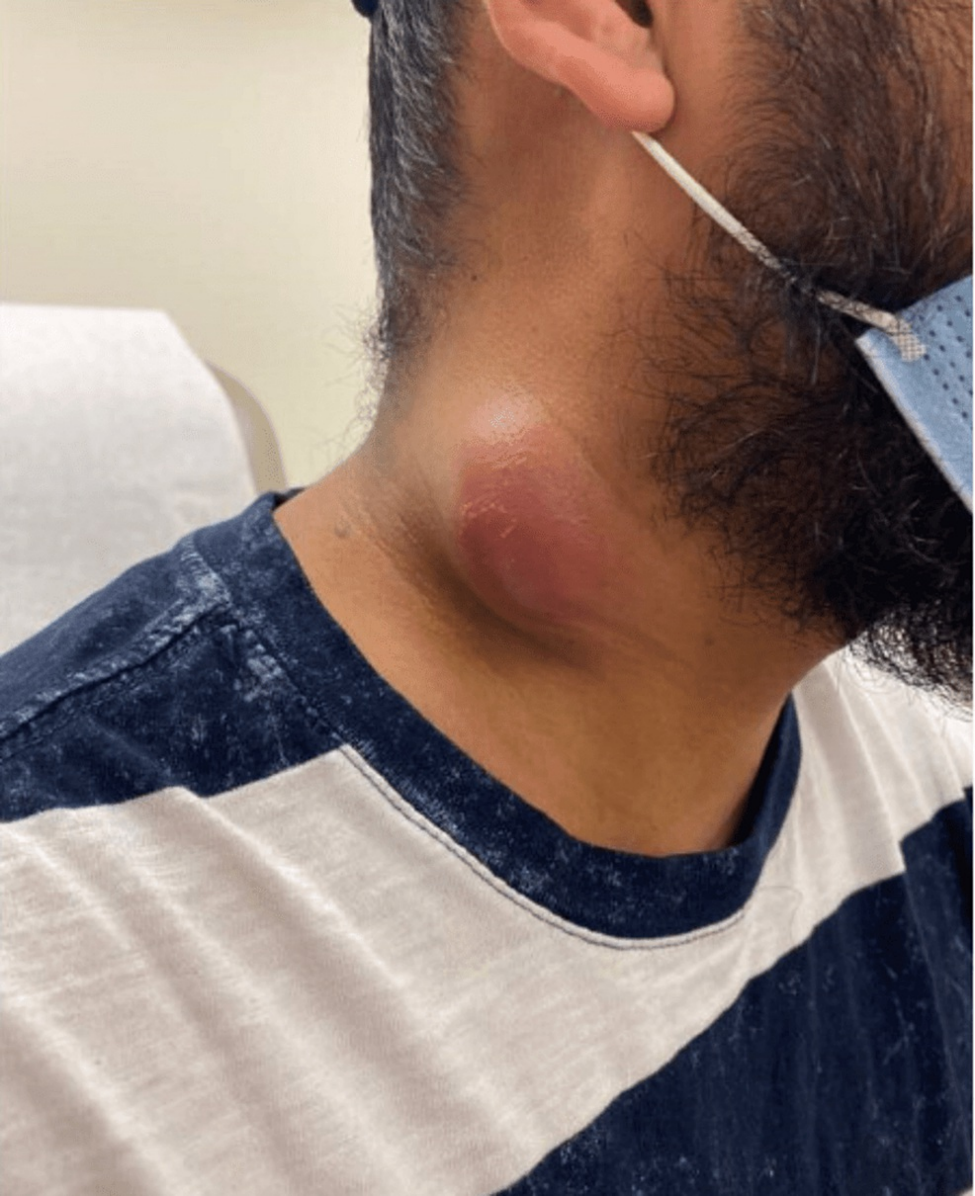
Erythematous right neck lump

A biopsy (Figure 3) revealed chronic inflammation without evidence of malignancy. Quantiferon TB test was positive with a TB-Nil value of 4.22 IU/mL. Samples for whole-genome sequencing were collected and sent to the University of Washington pathology department. Of note, mycoplasma PCR as well as Histoplasma and Bartonella cultures were negative. The patient reported little to no change in the size of the mass (Figure 4) at this time and was scheduled to follow up in a couple of weeks to allow cultures to grow. The initial AFB culture eventually grew *Mycobacterium tuberculosis* and anti-tubercular therapy was initiated with rifampin, isoniazid, pyrazinamide, and ethambutol (RIPE) along with vitamin B6. Two weeks later, the organism was proven to be pan-susceptible.

**Figure 5:**
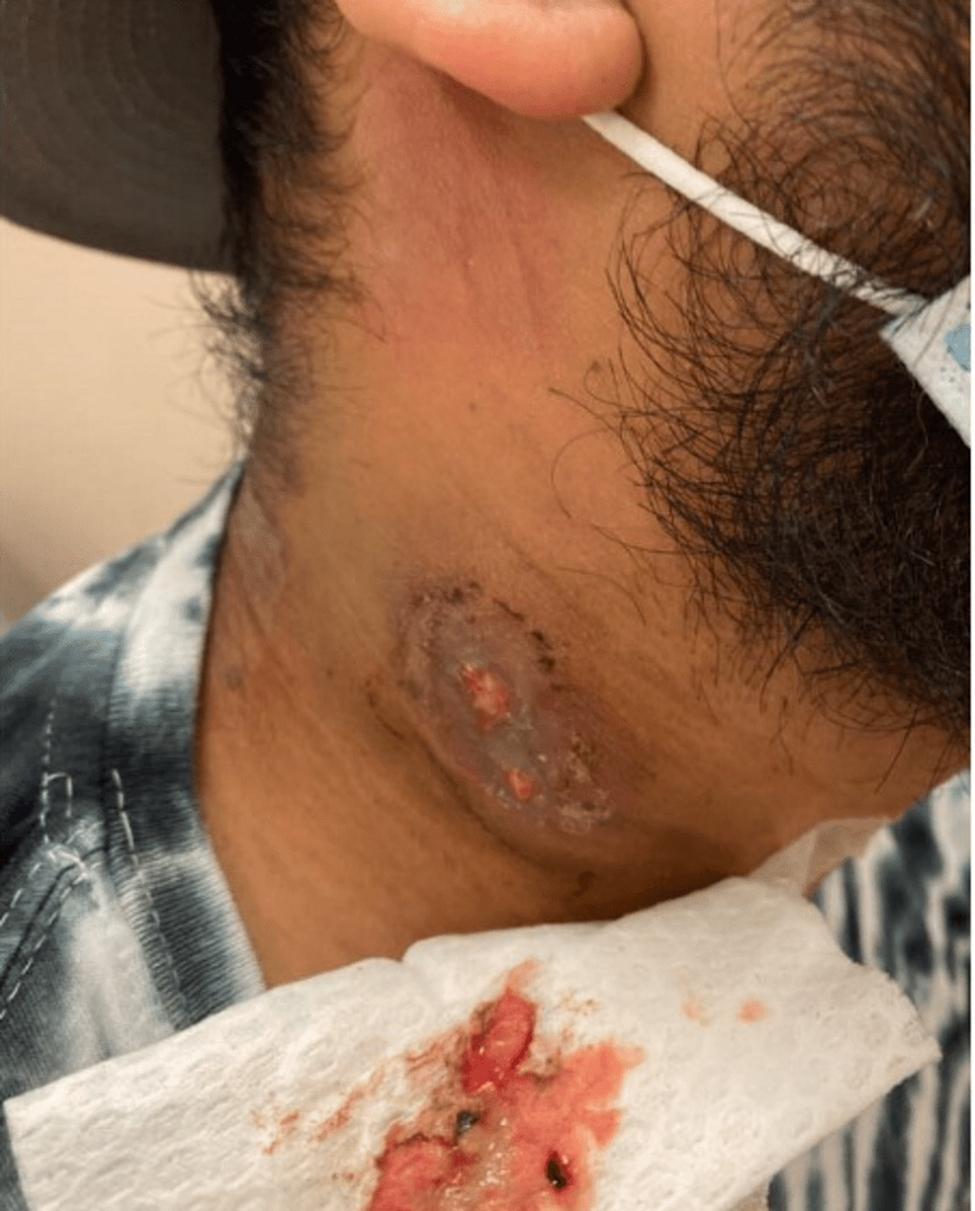
Suppurating wound one week after starting RIPE therapy RIPE, rifampin, isoniazid, pyrazinamide, and ethambutol.

**Figure 6:**
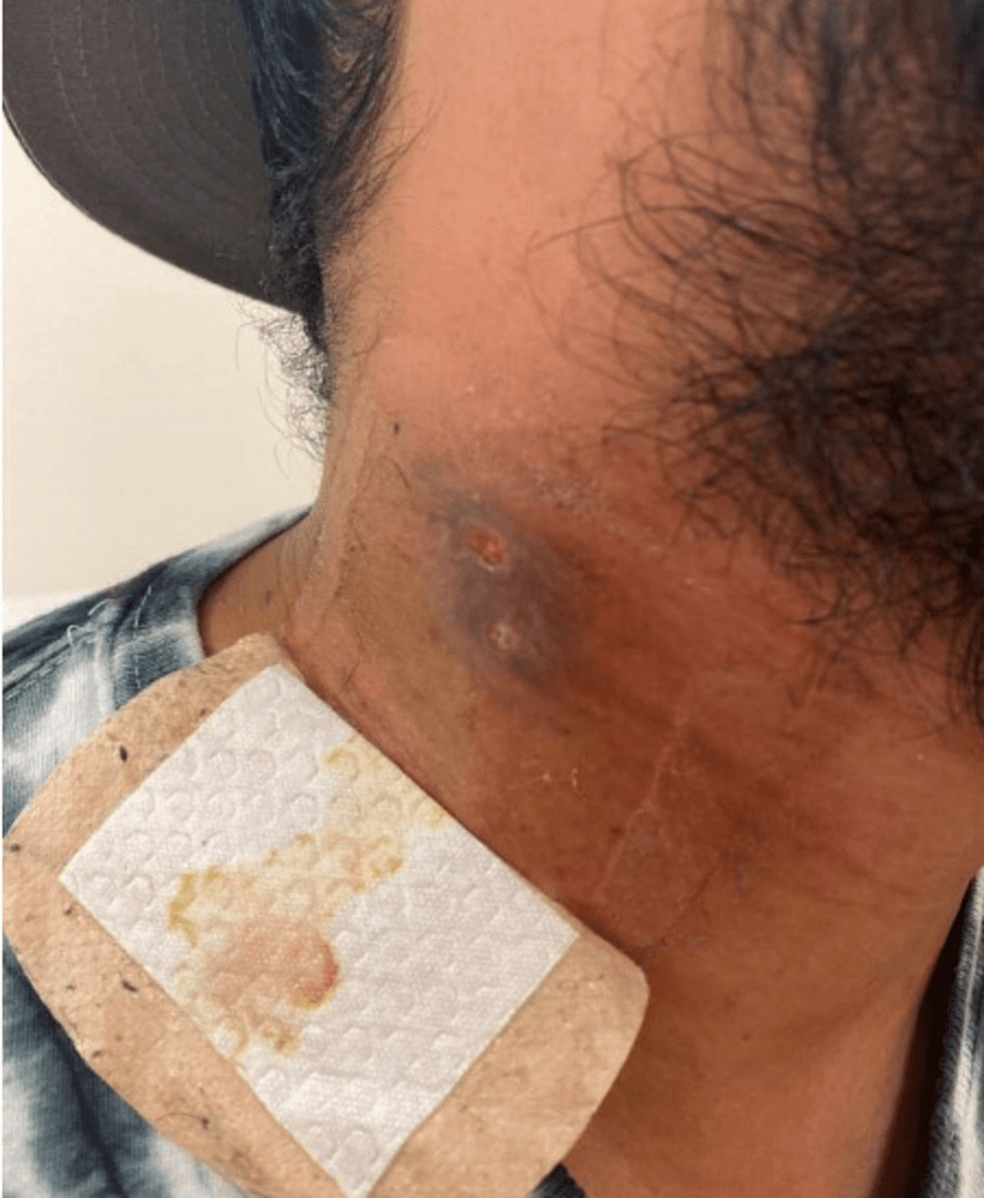
Wound with residual serosanguinous drainage

One week after initiation of RIPE therapy, the neck mass suppurated serosanguinous fluid (Figure 5). The patient reported improving symptoms with some lingering fatigue. Repeat blood work was completed, including a CBC demonstrating normal white blood cell level with a relative lymphocytosis (51.2%) and a hepatic function panel with normalized AST (24 U/L) and ALT (48 U/L) levels - an improvement from prior studies. One month later, he exhibited a drained wound (Figure 6) and endorsed near-complete symptom resolution. Ethambutol was discontinued one month after initiation.

**Figure 7:**
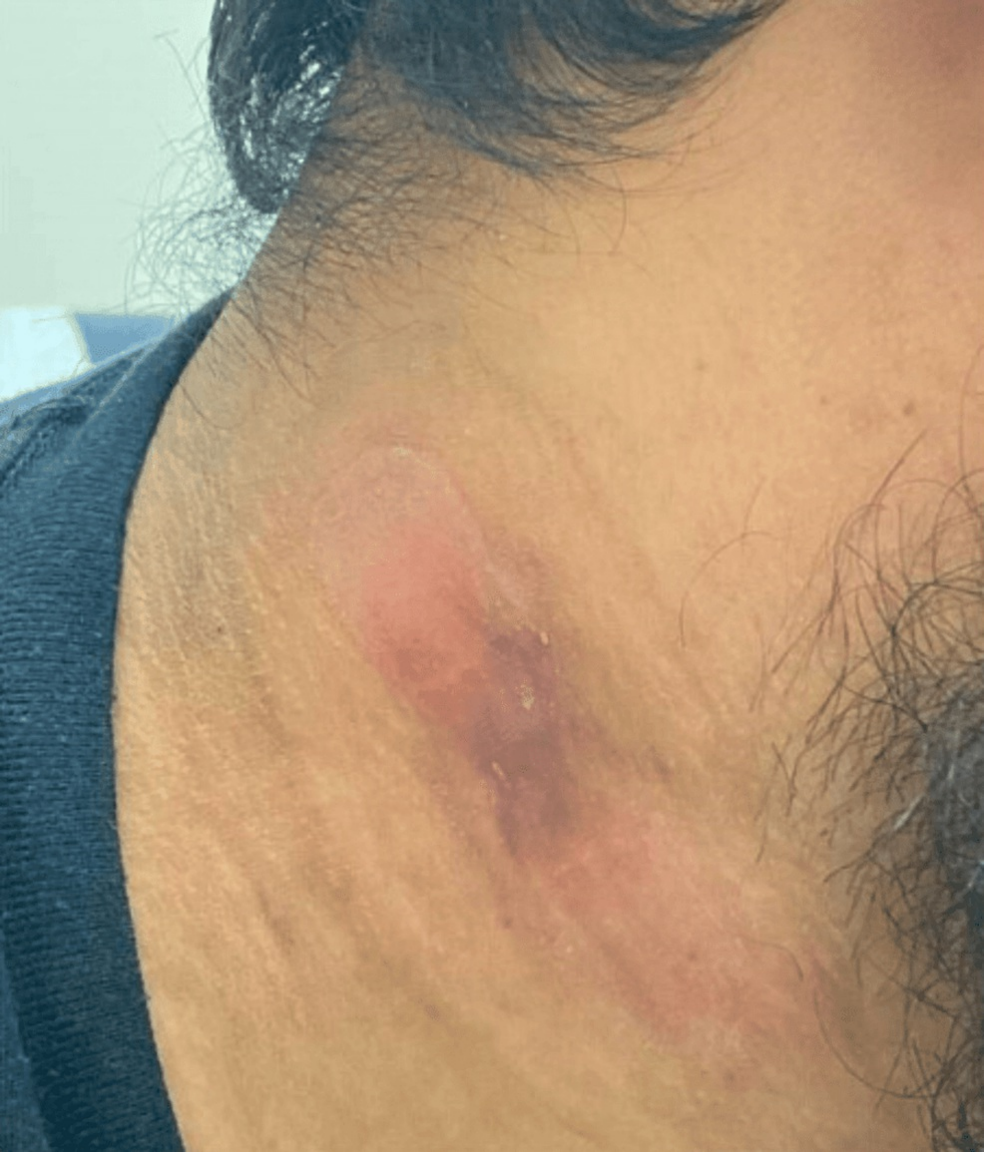
Healing neck wound

**Figure 8:**
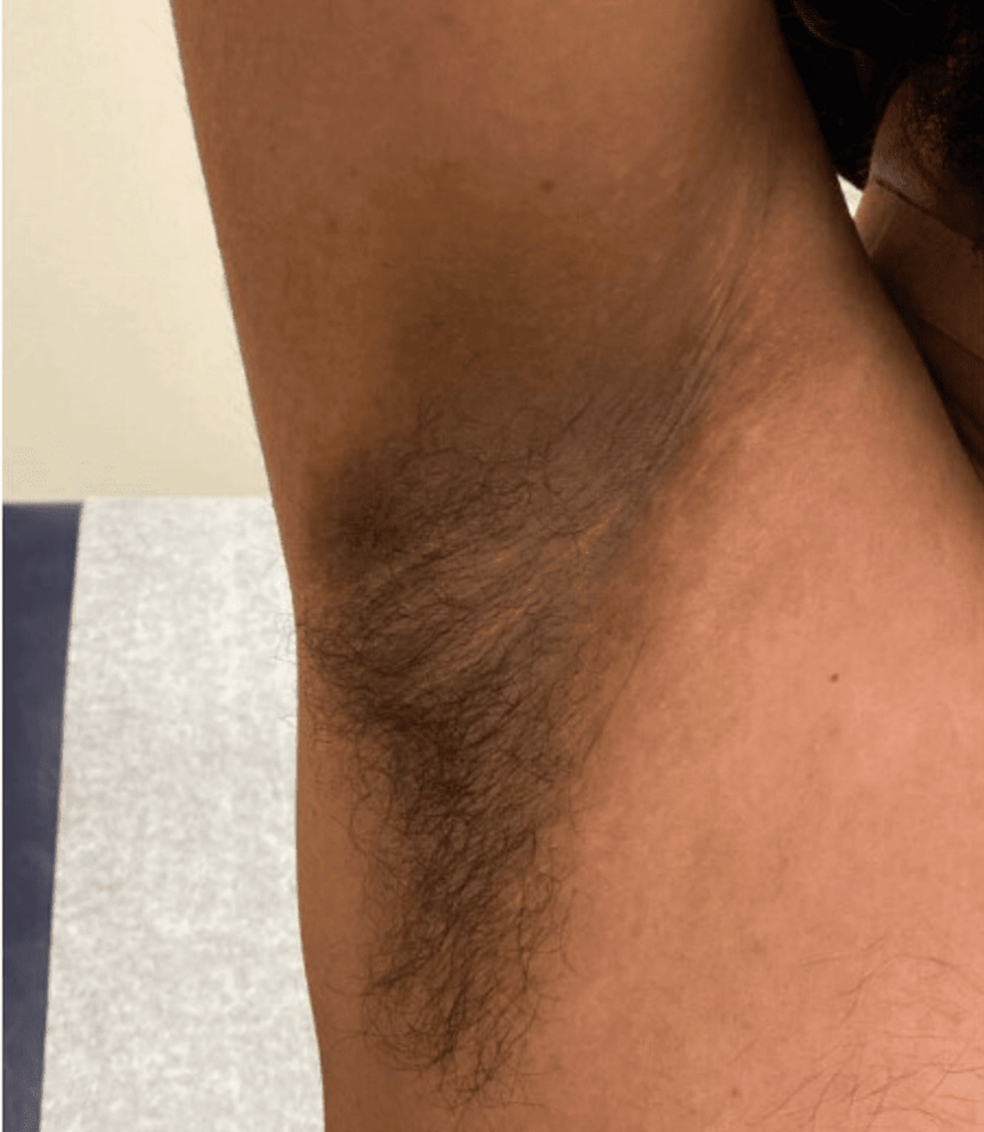
New-onset acanthosis nigricans in the axilla

**Figure 9:**
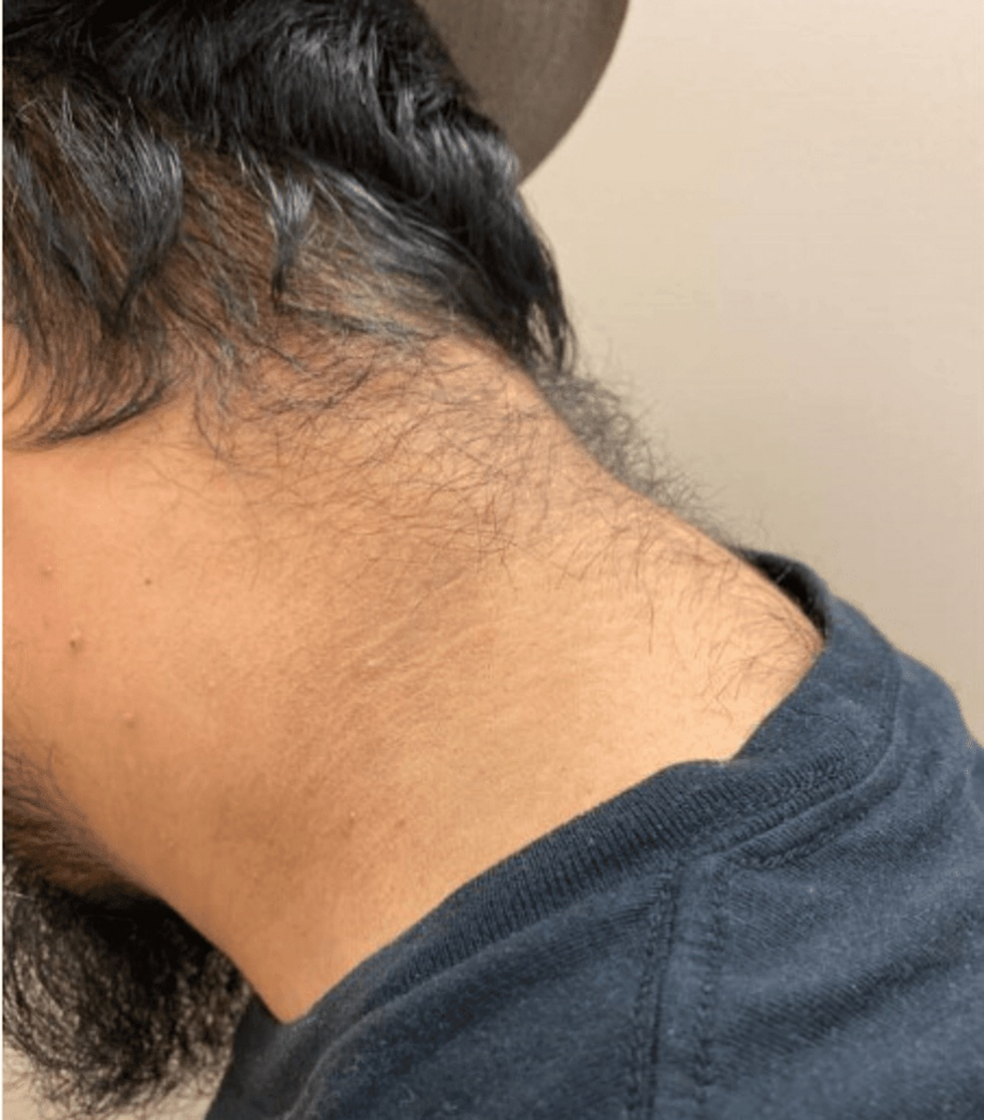
New-onset acanthosis nigricans on the neck

Pyrazinamide was discontinued after two months. Isoniazid and rifampin, along with vitamin B6, were continued. At this time, the wound appeared to be healing well (Figure 7), but the patient reported weight gain, increased appetite, and new skin changes in both the axilla and on the neck, consistent with AN (Figures 8, 9). There was no family or personal history of diabetes, his HbA1c was 5.4%, and CMP and liver enzymes were within normal limits.

**Figure 10:**
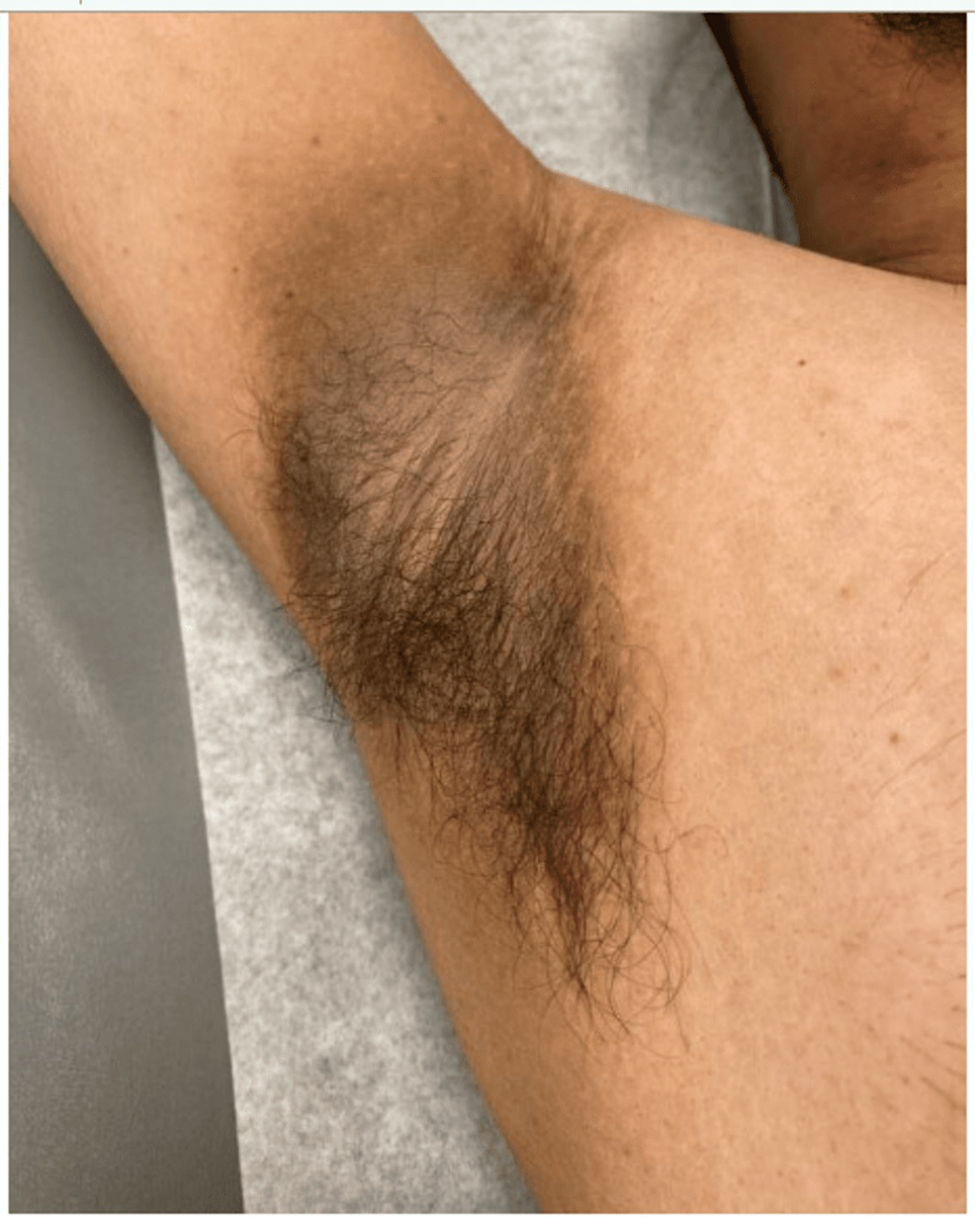
Acanthosis nigricans on the axilla one month after onset

**Figure 11:**
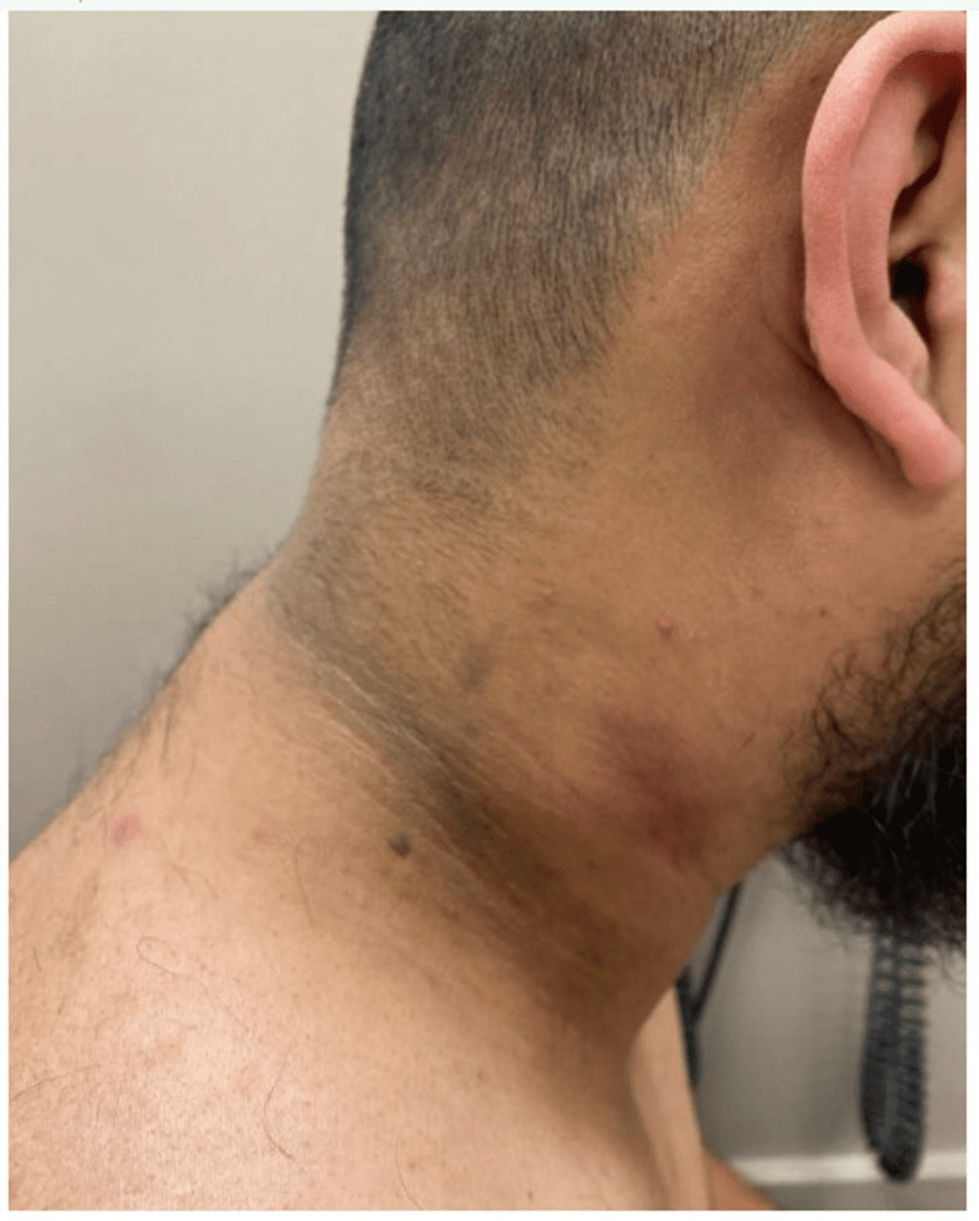
Acanthosis nigricans on the neck one month after onset and neck wound progress at four months of treatment

A referral was sent to gastroenterology to screen for gastric cancer presenting as new-onset AN. A follow-up esophagogastroduodenoscopy was unremarkable. It was determined that the skin discoloration was likely due to isoniazid therapy and the patient was recommended to continue treatment as planned due to the benign nature of the finding. The patient’s AN persisted four months after initiation of treatment and remained unresolved at the time of submission (Figures 10, 11). 

## Discussion

In adults, 95% of mycobacterial cervical infections are caused by *M. tuberculosis*, with the other 5% attributed to atypical mycobacteria such as *M. scrofulaceum*, *M. avium*, and *M. haemophilum* [6,7]. Extrapulmonary manifestations are common, occurring in 15-30% of all cases of pulmonary TB [8]. These findings include migration to the oropharynx, larynx, ear, salivary glands, nose, and lymph nodes [9]. Lymph node TB is one of the most common extrapulmonary presentations of the disease, especially in countries in which TB is not endemic [10]. Of these cases, 60-90% affect the cervical lymph nodes, resulting in CTL [10]. While CTL is thought to occur secondary to pulmonary TB dissemination to the cervical region via lymphatics, multiple reports show that it can present as a primary lymphatic infection in the absence of pulmonary disease [10]. In fact, the majority of scrofula cases occur in the absence of pulmonary TB [9]. 

CTL usually presents as a painless, enlarging neck mass with an average size of 3 cm and duration of three to four weeks, but has been reported to last up to eight months [9]. It most commonly affects the posterior cervical and supraclavicular nodes [9]. Two-thirds of all patients have multiple masses and one-third of all patients have bilateral masses [7]. Nearly half of the patients report fever, chills, weight loss, or malaise [7]. Other symptoms that may be observed include night sweats, cough, and weakness [10]. Complications include the formation of fistulas or abscesses, such as in this patient, and rarely, life-threatening hematogenous dissemination of the bacteria, known as miliary TB [11]. While neck abscesses due to staphylococcal and streptococcal species may become life-threatening, TB abscesses, termed “cold abscesses”, are typically indolent [9]. 

Scrofula is observed more frequently in females between the ages of 30 and 40 years, likely due to heightened body awareness and worsened living conditions [10]. Endemic areas for infection include Sub-Saharan Africa and Asia, especially India [8]. In Nepal, the country of origin of our patient, drug-resistant TB is among the top 10 causes of death, leading to approximately 17,000 deaths a year [12]. 

The differential diagnosis for a neck mass should include mycobacterial cervical lymphadenitis, which encompasses TB and non-tuberculous mycobacteria sarcoidosis, toxoplasmosis, tularemia, fungal infections cat-scratch disease, and cancer [6]. When working up CTL, purified protein derivative testing or interferon-gamma assay may be suggestive of prior TB exposure but are not diagnostic of active TB [11]. CBC, CMP, inflammatory markers, and imaging modalities such as CT, magnetic resonance imaging (MRI), and ultrasound are non-diagnostic but may enhance clinical suspicion and rule out other pathology. Non-specific findings include leukocytosis, thrombocytosis, anemia, hyponatremia, and elevated erythrocyte sedimentation rate [6]. While fine-needle aspiration of the mass is highly specific and less invasive, it is only moderately sensitive. Excisional biopsy has a higher sensitivity and is the definitive test for diagnosing CTL [11]. AFB culture has the highest sensitivity and specificity but takes time, and molecular methods may provide earlier answers to diagnostic dilemmas. When cultures remain negative, clinical judgment based on a patient's history, exposures, physical examination findings, laboratory testing, and imaging results should be used to diagnose CTL [7]. All patients with suspected CTL should be tested for HIV due to higher rates of association between these two conditions [6].

CTL is treated empirically with two months of RIPE followed by four months of rifampin and isoniazid - collectively known as the RIPE regimen [11]. While 85% of TB cases can be cured with treatment, mortality may reach up to 50% when the disease is untreated [1]. Treatment may need to be modified when AFB culture reveals drug resistance. Non-RIPE interventions for scrofula include surgery, steroids, and preventative measures; however, incision and drainage alone is ineffective due to high recurrence rates [7]. Surgical excision may be effective in immunocompetent patients with isolated CTL, but the risks include fistulization and hematogenous spread of bacteria [11]. Corticosteroids have an unclear efficacy and are usually reserved for patients in severe discomfort or patients who exhibit paradoxical reactions which is characterized by worsening of symptoms on therapy due to immune response to antigen release by dying organisms [11]. Some endemic regions implement the Bacille Calmette Guerin (BCG) vaccine, which is especially effective at preventing TB in infants and children [13]. The prognosis for scrofula is favorable when identified early and treated adequately, but the prognosis is worsened when scrofula is incorrectly diagnosed and treatment is delayed [11]. 

AFB culture showed our patient’s strain of TB to have no drug resistance, and his HIV screen was negative, so he was promptly started on the RIPE regimen, along with vitamin B6. This regimen can elicit various side effects, most notably drug-induced liver injury, neuropathy secondary to isoniazid-induced vitamin B6 deficiency, and dose-dependent optic neuritis from ethambutol [14]. As such, vitamin B6 is often used as adjunct therapy in patients treated with RIPE, and patients are instructed to receive vision screening before and during RIPE treatment [14]. Due to variations in isoniazid metabolism via N-acetylation, side effects can vary significantly from person to person; thus, “slow acetylators” may be excessively prone to adverse reactions [14]. Rifampin may induce hyperbilirubinemia, transaminitis, flu-like syndrome, respiratory distress, hemolytic anemia, leukopenia, thrombocytopenia, and drug-drug interactions due to being a cytochrome inducer [14]. Pyrazinamide is the most common offender in eliciting adverse drug events, which include hepatotoxicity and gastrointestinal upset, and thus is typically avoided in the elderly [15].

Interestingly, our patient presented with signs of AN following the initiation of RIPE therapy. AN is most frequently observed in obesity and states of insulin resistance, such as type 2 diabetes mellitus (T2DM) but may also be elicited secondary to medication side effects [16]. The pathophysiology of AN is the proliferation of the epidermal keratinocytes and dermal fibroblasts, which occurs via activation of the insulin-like growth factor-1 receptor (IGF1-R), fibroblast growth factor receptor, and epidermal growth factor receptor [5]. T2DM and obesity are states of hyperinsulinemia, and insulin is known to directly activate IGF1-R on epidermal cells and increase IGF1 levels in the blood, which is the proposed mechanism of action of AN in hyperinsulinemic states [5]. AN usually resolves following weight loss, adequate control of T2DM, and/or discontinuation of an offending medication [5]. 

There have been few papers outlining the influence of RIPE therapy on glycemic regulation and insulin sensitivity. Waterhouse et al. presented a case report in 2005 detailing new-onset insulin-dependent diabetes in a woman treated with RIPE therapy which subsequently resolved after discontinuation of treatment [17]. Another report from 2015 by Manish et al. outlined the development of drug-induced diabetes in a six-year-old patient receiving isoniazid treatment for pulmonary TB [18]. A 1953 prospective cohort study by Luntz and Smith showed evidence that diabetics taking isoniazid require increased dosing of insulin [19]. It is also well known that TB and diabetes are mutually inducive, likely due to T-cell and cytokine alterations [20]. Mechanistically, it is plausible that isoniazid could induce diabetes and hyperinsulinemia, which may have subsequently caused this patient’s AN. However, we found no study reporting the incidence of AN during RIPE therapy, nor specifically as an adverse reaction to isoniazid. Our patient showed no personal or family history of diabetes and had a normal HbA1c level. Although this side effect is thought to be benign, documenting it in the literature is critical for future patient outcomes as well as providing further elucidation of the potential adverse effects of medications.

## Conclusions

The presentation of scrofula without pulmonary involvement, as observed in this patient, serves as a reminder of the diverse ways in which TB can manifest, necessitating a thorough differential diagnosis when evaluating suspicious lymphadenopathies. Special attention should be allocated to patients originating from or having recently visited endemic regions in hopes of reducing morbidity and mortality of the disease. Moreover, the management challenges, including the initiation of appropriate antitubercular therapy, monitoring for medication-related adverse effects, and the potential need for surgical intervention, offer valuable insights into treating CTL. 

Isoniazid-induced AN is a previously unreported adverse reaction to standard RIPE therapy. The development of novel side effects in a longstanding treatment regimen emphasizes the need for further research and awareness regarding atypical presentations and management nuances of extrapulmonary TB.
